# Presence of asymptomatic Peripheral Arterial Disease in combination with common risk factors elevates the cardiovascular risk Substantially

**DOI:** 10.1016/j.ijcrp.2022.200130

**Published:** 2022-04-18

**Authors:** Fredrik Sartipy, Antonio José Garcia Pereira Filho, Fredrik Lundin, Eric Wahlberg, Birgitta Sigvant

**Affiliations:** a Section of Vascular Surgery, Department of Clinical Science and Education, Karolinska Institutet at Södersjukhuset, Kirurgkliniken, Sjukhusbacken 10, 118 83, Stockholm, Sweden; b Department of Surgical Sciences, Uppsala University, Uppsala, Sweden; c Centre of Clinical Research, County Council of Värmland, Älvgatan 49, 652 30, Karlstad, Sweden; d Department of Medicine and Health, Linköping University, Linkoping University Hospital, SE 581-53, Linköping, Sweden

**Keywords:** Risk factor, Ankle-brachial index, Cardiovascular risk, APAD, Asymptomatic Peripheral Arterial Disease, CV, Cardiovascular, ABI, Ankle Brachial Index

## Abstract

**Background and aims:**

This study evaluates the risks for adverse cardiovascular (CV) events in Asymptomatic Peripheral Arterial Disease (APAD) combined with different traditional CV risk factors.

**Methods:**

A population-based observational study of 8000 subjects, identified 559 subjects as having APAD through ankle-brachial index (ABI) measurements and questionnaires regarding limb symptoms. This cohort and subgroups classified by presence of different traditional CV risk factors at baseline were assessed for 10 years on CV outcome. The recorded endpoints were all-cause mortality, CV mortality and non-fatal CV events.

**Results:**

Before subdividing the APAD subjects, the CV mortality incidence was 28.5 deaths per 1000 person-years as compared to 8.7 deaths for references without APAD. For subjects with hypertension at baseline the CV mortality incidence was 35.4 when combined with APAD and 11.7 without. In women with hypertension but without other risk factors, presence of APAD increased the age-adjusted Hazard Ratio (HR) for fatal and non-fatal CV events by 1.86 [CI 1.54,2.24, p < 0.001].

**Conclusions:**

ABI measurements should be considered an important indication for aggressive multifactorial risk factor reduction in populations with any other prevalent CV risk factor. In hypertension, diabetes mellitus and a smoking history, coexisting APAD contributes significantly to the increased age-adjusted CV risk.

## Disclosures

The original study was supported by unrestricted grants from The 10.13039/501100003793Swedish Heart-Lung Foundation, Värmland's County Research Council, and Sanofi-Aventis® and Astra Zeneca®.

## 1 Introduction

One trend in medicine today is to move focus from treatment of established disease to prevention [1]. The rationale is that the costs for chronic conditions are expected to rise and prevention strategies may improve health care [2] and save resources [3]. One such chronic disease is Peripheral Arterial Disease (PAD), which in its’ earliest stage asymptomatic PAD (APAD) is easily detected by a simple and reliable Ankle-Brachial Index (ABI) measurement [4]. APAD is common among elderly and associated with increased risk for cardiovascular (CV) morbidity and mortality [[5], [6], [7], [8]].

While there is a lack of up to date epidemiological data about the APAD group [8,9], we have previously shown that up to one third of APAD subjects has manifestations of poly-vascular disease [10]. Therefore, it is reasonable to believe that within the APAD population there are subjects with a wide range of arteriosclerotic burden and risks, some particularly suitable for preventive interventions [11,12]. So far the benefits of life style measurements and medical prophylactic treatment in APAD are insufficiently validated [[13], [14], [15], [16]], but screening for certain APAD high-risk populations and intensified prevention of CV risk in these may diminish morbidity and a improve cost-effectiveness [14].

The aim of this study was to identify subgroups of APAD with high risk for CV events. To accomplish this, we chose to assess CV long term outcome in APAD subgroups classified by presence of traditional CV risk factors at base line. The importance of the study was to identify subgroups of APAD particularly suitable for further studies of screening for APAD and subsequent treatment.

## 2 Methods

### 2.1 Cohort and data collection

The study population and data collection process for the 10-years follow-up was described in detail 2007 and 2019 as the Swedish Study of Prevalence of PAD in Society [10,17], why only a brief summary is presented here. The cohort was population-based and covered subjects selected through randomization of Swedish inhabitants aged 60–90 years in 2004. To cover demographic and geographic distributions in the Swedish population, four separate regions in the country were selected. A random sample from these regions consisting of 2000 subjects from each region, was extracted from the government's tax register in June 2004. Data was collected through physical examinations and questionnaires (the Rose's World Health Organization questionnaire and Walking Impairment Questionnaire [18,19]) at baseline. Outcome information was complemented with data from validated Swedish national health registers. Subjects who had a brachial blood pressure above 180 mmHg were referred to their general practitioner, but no other intervention was made during the assessment period. The observation time in this study was from autumn of 2005 until end of year 2015.

### 2.2 Definitions

APAD was defined by an ABI<0.9 without qualifying answers for symptomatic PAD in the questionnaires. Subjects with an ABI<0.9 and qualifying answers for symptomatic PAD were excluded. Subjects with an ABI≥0.9 served as references (REF).

ABI was determined by measuring the blood pressure in the right arm using a 12-cm-wide blood pressure cuff with the patient in a sitting position. The ankle blood pressures were measured using a pocket CW-Doppler (8-MHz Doppler-probe, Hadeco, Sweden) and the same blood pressure cuff. Measurements were performed twice in each limb by insonating both the posterior tibial and dorsal pedal arteries by specially trained nurses [17]. The threshold value of <0.9 was chosen according to the American Heart Association's Scientific Statement and the European Society of Vascular Surgery guidelines [20,21]. Information on the following risk factors was obtained from registers (cardiac disease included ICD-10 codes: I20, I21, I50, diabetes: E10–14, stroke/TIA: I60–69, and hypertension: I10–15) combined with self-reported data in health questionnaires. Prevalence of any of these conditions was defined by either presence of these ICD-codes in the registers or as self-reported information. History of smoking was defined as any self-reported smoking activity.

The APAD group was divided into subgroups defined by presence of any of the following risk factors at inclusion; cardiac disease, stroke/TIA, diabetes, hypertension or smoking history. In each of these groups other comorbidity was not excluded. Therefore, three additional subgroups were also generated: Subgroup of hypertension only (i.e. subjects without other risk factors than hypertension at base line), Subgroup of diabetes only (i.e. subjects without other risk factors than diabetes at base line), and the Subgroup with exclusion of cardiac disease or stroke/TIA.

Pharmacological treatment at inclusion was also collected as self-reported information, completed with data from the Prescribed Drug Register by 2004–05. Any antiplatelet or anticoagulation, antihypertensive, statin, and diabetic therapy were recorded. Classification used was according to the following ATC-codes; for antiplatelet and anticoagulation therapy B01AC, B01AA, B01AB, B01AE, B01AF and B01AX, statins C10AA and C10B, and antihypertensive treatment C02, C03, C07–C09. Drugs dispended from pharmacy were used as a proxy for drug usage.

Primary end-points were all-cause mortality, CV-mortality, non-fatal CV-events and the composite endpoint CV-mortality and non-fatal CV events, with the following ICD-10 codes for CV events: I10–15, I20–25, I50–51, I60–69 and I70–73.

### 2.3 Analyses

Baseline data was presented using mean and standard deviation (age, ABI, and BMI) and absolute and relative frequencies (comorbidities). Mortality and non-fatal events were described by absolute and relative frequencies, and incidence in number of events per 1000 person-years. The analysis was carried out defined by the presence or not of one of the following conditions at baseline: cardiac disease, stroke/TIA, diabetes mellitus, hypertension and smoking history. Mortality was presented as age-adjusted survival curves with p-values for differences between APAD and REFs and proportional hazards test for different risk factors. A regression model was performed to determine risk for events with group membership as the analysis variable, unadjusted and adjusted for age. Hazard Ratios (HR) for CV events for APAD overall and with different risk factors present was calculated by using a Royston-Parmar models (stata command stpm2) with three degrees of freedom for the baseline hazard and two degrees of freedom for the time-dependent effect of age. Tests of the proportional hazards assumption of the group variables were performed with graphical methods (Stata program stphplot) and showed no serious indication of non-proportionality. A Royston-Parmar models was also used to investigate event risk (HR) for APAD and REF with the same base-line risk factors adjusted for baseline age using a regression strategy, as described above. All comparisons were made with considerations of sex differences. All statistical analyses were carried out using Stata MP/4 ver. 16.1 (StataCorp LP, College Station, Texas) and statistical test were two-sided.

### Ethical approval

The study was approved by the local ethics committees in Stockholm (KI 03–538 and Dnr 2014/2070–32), Umeå University (Dnr 03–459), Lunds University (832–0), Uppsala University (Dnr 03–564) and Örebro (Dnr 374–03). Informed consent was obtained from each participant.

## 3 Results

### 3.1 Cohort

Of the original 8000 invited subjects, 5057 subjects accepted to participate in examinations. A total of 4659 subjects fulfilled the inclusion criteria and 559 (12%) were diagnosed as having APAD (Table 1) and 4100 (88%) as REF, with a higher prevalence of APAD among women (62% versus 38% for men). Some 398 subjects of the original 8000, were excluded from analyses as they were classified having symptomatic PAD. Among the included subjects, the mean age was 75.6 years (similar among men and women) as compared to 69.8 years for REF. In the APAD group presence of the comorbidities cardiac disease, diabetes, stroke and hypertension was significantly higher, as was having a smoking history. With exception for hypertension, men had higher CV-related comorbidity than women. Aside from statins, that were used by 18% of APAD subjects and 15% of the REF at base line, usage of CV protective drugs was clearly more common in APAD subjects. Antiplatelet therapy, anticoagulation and treatment for hypertension was used by 32.7%, 6.3% and 54.2%, respectively, in APAD subjects and 20.6%, 3.2% and 37.5% in the reference population.

**Table 1:** Baseline Characteristics of the Study Cohort 2005 by sex (p-values refers to comparisons between References and the Asymptomatic PAD group).

Variables	Sex	References	Asymptomatic PAD	p-value
**Group size, N (%)**	All	4100	559	
	Men	1908 (46.5)	211 (37.7)	
	Women	2192 (53.5)	348 (62.3)	
**Age, mean (SD)**	All	69.8 (7.5)	75.6 (8.0)	<0.001
	Men	69.5 (7.4)	75.4 (8.0)	<0.001
	Women	70.1 (7.6)	75.8 (8.1)	<0.001
**Body Mass Index, mean (SD)**	All	26.2 (4.0)	25.5 (3.9)	<0.001
	Men	26.2 (3.6)	25.6 (3.1)	<0.001
	Women	26.2 (4.3)	25.4 (4.3)	<0.001
**Ankle-Brachial Index, mean (SD)**	All	1.05 (0.09)	0.78 (0.11)	<0.001
	Men	1.08 (0.10)	0.77 (0.10)	<0.001
	Women	1.03 (0.08)	0.78 (0.12)	<0.001
**Cardiac disease, N (%)**	All	720 (17.6)	149 (26.7)	<0.001
	Men	400 (21.0)	65 (30.8)	0.002
	Women	320 (14.6)	84 (24.1)	<0.001
**Diabetes mellitus, N (%)**	All	363 (8.9)	75 (13.4)	<0.001
	Men	182 (9.5)	34 (16.1)	0.005
	Women	181 (8.3)	41 (11.8)	0.040
**Stroke/TIA, N (%)**	All	248 (6.0)	79 (14.1)	<0.001
	Men	142 (7.4)	42 (19.9)	<0.001
	Women	106 (4.8)	37 (10.6)	<0.001
**Smoking history, N (%)**	All	2092 (51.0)	306 (54.7)	<0.001
	Men	1182 (61.9)	150 (71.1)	<0.001
	Women	910 (41.5)	156 (44.8)	<0.001
**Hypertension, N (%)**	All	1420 (34.6)	239 (42.8)	<0.001
	Men	629 (33.0)	81 (38.4)	0.124
	Women	791 (36.1)	158 (45.4)	0.001

Abbreviations used: PAD (Peripheral Arterial Disease), N (Numbers), SD (Standard Deviation), TIA (Transient Ischemic Attack).

### 3.2 All-cause mortality

During the observation period 56.2% of APAD subjects and 27.4% of REF died (Table 2). After adjustments for age and baseline comorbidity the HR for all-cause mortality was 1.53 [CI 1.34,1.75, p < 0.001] for APAD. The highest all-cause mortality was seen in men with APAD having a history of stroke/TIA or cardiac disease, 78.6% and 75.4%, respectively. These two groups also had the highest incidence of non-fatal CV events. Women with APAD were slightly less likely to suffer from any event than men, except for women with diabetes who had a higher all-cause mortality than men, 73% versus 59%.

**Table 2:** Incidence in mortality and non-fatal cardiovascular events for subjects with Asymptomatic Peripheral Arterial Disease (APAD) and References (REF) separated by risk factors at baseline.

	Group	Subjects, N (%)	All-cause Mortality		Cardiovascular Mortality		Non-fatal Cardiovascular events	
			N (%)	Incidence	N (%)	Incidence	N (mean)	Incidence
**Total group**	REF	4100 (100.0)	1125 (27.4)	27.7	353 (8.6)	8.7	7963 (1.9)	196.1
	APAD	559 (100.0)	314 (56.2)	67.8	132 (23.6)	28.5	1582 (2.8)	341.5
**Prevalent risk factor at baseline**								
**Cardiac disease**	REF	720 (100.0)	301 (41.8)	46.3	127 (17.6)	19.5	3626 (5.0)	557.6
	APAD	149 (100.0)	111 (74.5)	112.6	51 (34.2)	51.8	672 (4.5)	681.8
**Stroke/TIA**	REF	248 (100.0)	108 (43.5)	48.3	52 (21.0)	23.3	920 (3.7)	411.6
	APAD	79 (100.0)	58 (73.4)	107.2	35 (44.3)	64.7	287 (3.6)	530.6
**Diabetes**	REF	363 (100.0)	146 (40.2)	43.7	48 (13.2)	14.4	1083 (3.0)	323.9
	APAD	75 (100.0)	50 (66.7)	86.3	20 (26.7)	34.5	339 (4.5)	585.3
**Hypertension**	REF	1420 (100.0)	438 (30.8)	31.7	162 (11.4)	11.7	3350 (2.4)	242.7
	APAD	239 (100.0)	146 (61.1)	76.0	68 (28.5)	35.4	659 (2.8)	343.1
**Smoking history**	REF	2092 (100.0)	560 (26.8)	27.0	159 (7.6)	7.7	4570 (2.2)	220.3
	APAD	306 (100.0)	169 (55.2)	67.5	72 (23.5)	28.7	998 (3.3)	398.4

Abbreviations used: APAD (Asymptomatic Peripheral Arterial Disease), REF (References), N (Numbers), TIA (Transient Ischemic Attack).

### 3.3 CV mortality and non-fatal events

After adjustments for age and baseline comorbidities, the HR for CV mortality was 1.80 [1.45, 2.22, p < 0.001] for APAD. The incidence of CV mortality was 28.5 deaths per 1000 person-years for APAD subjects as compared to 8.7 for REF. Men with APAD and previous stroke/TIA displayed the highest incidence (86.0 deaths per 1000 person-years), followed by men with cardiac disease (53.5). Corresponding incidences for REF were lower (28.5 and 24.2, respectively). The incidence of CV mortality among APAD females with stroke/TIA was 47.2, versus 16.9 for REF. Women with APAD and cardiac disease had 3.5 times higher CV mortality incidence as compared to the REF group (50.6 vs 14.0).

The incidence of non-fatal CV events was 341.5 events per 1000 person-years for the APAD group versus 196.0 for REF. Men in all subgroups, and particularly men with concomitant CV diseases, suffered from more non-fatal CV events as compared women.

### 3.4 CV risk for APAD subgroups compared with the entire REF population

Over the 10 years follow-up APAD resulted in an increased risk for a CV event in both the crude and the age-adjusted model. The highest risks were seen in APAD subgroups with either cardiac disease or diabetes (age-adjusted HR 2.54 [2.31–2.78] and 2.57 [2.28–2.89], respectively), which also was most prominent in women (HR 2.82 and 3.03) (Table 3). Having a smoking history almost doubled the risk (HR 1.86). Similar results were found for men and woman with hypertension (HR 1.57 and 1.81 respectively).

**Table 3:** Hazard ratio (HR) for fatal and non-fatal Cardiovascular events for men and women with Asymptomatic Peripheral Arterial Disease (APAD) separated by different risk factors at baseline (p-values refers to comparisons between References and the Asymptomatic PAD total group and subgroups).

Subgroup defined by prevalent risk factor by 2005	Sex	Subjects	Crude HR [95% CI]	p-value	Age-adjusted HR [95% CI]	p-value
**References**	All	4100 (100.0)				
	Men	1908 (46.5)				
	Women	2192 (53.5)				
**APAD (total)**	All	559 (100.0)	1.82 [1.72,1.93]	<0.001	1.48 [1.39,1.57]	<0.001
	Men	211 (37.7)	1.84 [1.69,2.00]	<0.001	1.54 [1.41,1.68]	<0.001
	Women	348 (62.3)	2.11 [1.94,2.30]	<0.001	1.60 [1.47,1.75]	<0.001
**APAD + Cardiac disease**	All	149 (100.0)	3.47 [3.17,3.79]	<0.001	2.54 [2.31,2.78]	<0.001
	Men	65 (43.6)	2.92 [2.57,3.32]	<0.001	2.38 [2.09,2.72]	<0.001
	Women	84 (56.4)	4.55 [4.02,5.15]	<0.001	2.82 [2.47,3.22]	<0.001
**APAD + Stroke/TIA**	All	79 (100.0)	2.96 [2.61,3.37]	<0.001	2.17 [1.91,2.48]	<0.001
	Men	42 (53.2)	3.09 [2.63,3.62]	<0.001	2.44 [2.08,2.87]	<0.001
	Women	37 (46.8)	2.80 [2.26,3.47]	<0.001	1.76 [1.42,2.19]	<0.001
**APAD + Diabetes**	All	75 (100.0)	3.14 [2.79,3.53]	<0.001	2.57 [2.28,2.89]	<0.001
	Men	34 (45.3)	2.50 [2.11,2.96]	<0.001	2.26 [1.91,2.68]	<0.001
	Women	41 (54.7)	4.26 [3.61,5.03]	<0.001	3.03 [2.56,3.58]	<0.001
**APAD + Hypertension**	All	239 (100.0)	1.96 [1.80,2.13]	<0.001	1.55 [1.42,1.69]	<0.001
	Men	81 (33.9)	1.87 [1.64,2.13]	<0.001	1.57 [1.38,1.80]	<0.001
	Women	158 (66.1)	2.48 [2.23,2.77]	<0.001	1.81 [1.62,2.03]	<0.001
**APAD + Smoking history**	All	306 (100.0)	2.10 [1.95,2.25]	<0.001	1.86 [1.73,2.00]	<0.001
	Men	150 (49.0)	2.16 [1.97,2.37]	<0.001	1.91 [1.74,2.09]	<0.001
	Women	156 (51.0)	2.08 [1.86,2.34]	<0.001	1.81 [1.61,2.03]	<0.001

Abbreviations used: APAD (Asymptomatic Peripheral Arterial Disease), N (Numbers), HR (Hazard Ratio), CI (Confidence Interval), TIA (Transient Ischemic Attack).

### 3.5 CV-risk comparisons between APAD subgroups and REF with the same risk factor

**Fig. 1:**
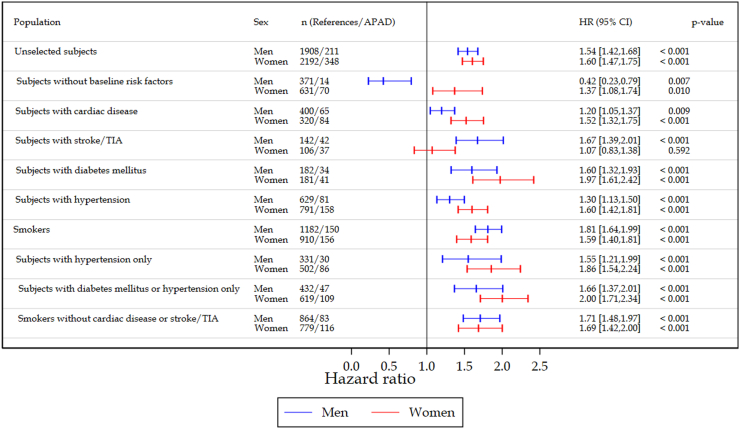
Age-adjusted Hazard Ratio (HR) for fatal and non-fatal Cardiovascular events for men and women with Asymptomatic Peripheral Arterial Disease (APAD) with the same base-line risk factors.

Overall, presence of APAD in combination with other CV morbidities or a smoking history increased the CV event risk (Fig. 1) and this additional CV-risk was more prominent in women than in men. The strongest impact of having the added diagnosis of APAD was observed in patients with either diabetes mellitus, hypertension or a smoking history. For example, male subjects with a smoking history of APAD contributed to an increased age-adjusted CV event risk by a HR of 1.81 [1.64–1.99]. In women with hypertension but without other risk factors, presence of APAD increases the HR by 1.86. For subjects without risk factors at base line APAD men had a lower risk for an event, with a HR of 0.42 [0.23–0.79] versus 1.37 [1.08–1.74] for women.

## 4 Discussion

First of all, this study confirms the diversity of CV risks among APAD subjects with different risk factors. Although general screening of APAD is not supported in the literature [22], finding APAD with ABI-measurements should be considered an important indication for aggressive multifactorial risk factor reduction in populations with any other prevalent CV risk factor.

This study's most important finding is that having APAD significantly increases the age-adjusted CV risk for patients with any of the traditional CV risk factors and especially for patients with hypertension, diabetes mellitus or those with a smoking history. This is particularly evident for women with diabetes and APAD, whose age-adjusted CV risk was increased by a factor of three.

It is well-known that patients with hypertension have an increased risk for CV morbidity, which motivates treatment [23]. In a large meta-analysis the risk for CV mortality in hypertension was determined to be doubled in a general cohort with the same age-range as the present [24], which is in accordance with the findings in this study. Similar results are found in subjects with diabetes. For example, diabetes in elderly is estimated to give an about two-fold excess of CV risk [25]. Assessing the impact of smoking history has proven more difficult because it is often combined with other risk factors. A recent study estimated that tobacco use resulted in more than a doubled CV risk [26]. The present cohort of APAD smokers without other risk factors had a 70% increased risk as compared to REF.

Despite advances in best medical treatment the overall mortality for APAD subjects in this study was similar to results presented over two decades ago [27,28]. More recent data has noted that the CV mortality in association with APAD is increased by a HR 1.93 [29] and HR 1.83 [7], which is similar to the results in the present study. Moreover, as in these studies, CV disease was the most common cause of death for APAD subjects. Among subjects without a comorbidity at baseline, CV risk was increased for women with APAD but not for men. This may be a finding by chance, as consequence of few subjects in this subgroup. It may, however, also indicate that lower extremity arteries could be an index site for arteriosclerosis in women while coronaries and cerebral arteries more often are afflicted in men [30].

In the literature there are examples of studies evaluating CV outcome for patients with traditional CV risk factors and a pathologic ABI. In such cohorts with cardiac disease and symptomatic PAD significantly elevated CV risks are presented [31,32] when compared to groups without PAD. In this study the group of APAD subjects with cardiac disease, the age-adjusted risk for CV events was increased by a factor of two and a half to three. Also in hypertensive patients the addition of symptomatic PAD increased the risk for adverse CV events [33]. The added CV risk of having a pathologic ABI was also assessed in patients with diabetes mellitus type 2 and noted as an odds ratio of 3.6 compared to those with diabetes and a normal ABI [34]. It is difficult to find outcome data in the literature on APAD and smoking in combination that is comparable to this cohort, but by large the risk magnitudes reported appear to be in line with the present data. For example, in a cohort of male smokers with an ABI≤0.9 the adjusted relative risk was 1.88 and only 1.08 for smokers with no PAD, both risks compared to groups of non-smokers without PAD [35]. In this study, subjects with a smoking history and an abnormal ABI had an almost doubled risk for death and CV-events as compared to REF.

Diagnosis of APAD may enable effective CV-event reduction. Measurement of ABI has been proven reliable for identification of PAD with a high specificity (83%–99%) but with a lower sensitivity (69–89%) [36,37]. Lipid lowering drugs are among the most cost-effective interventions available to health systems [38] and statins have been shown to reduce CV risks even in low-risk PAD populations [39,40]. So far CV event-reducing prophylaxis drug programs are under continuous development and adaptation but has not given attention to the increased risk APAD brings.

Current treatment recommendations for patients with hypertension or for smokers do not include statins, nor platelet inhibition, but may be expanded to include subjects with concomitant APAD. Accordingly, screening for APAD should be targeted subjects with hypertension and in those with a smoking history, followed by evaluation of broader prophylactic measures. To date, statins are recommended in APAD, even without comorbidities, but screening activities has not yet proven cost-effective. As CV risks are higher for hypertensive APAD subjects than for APAD alone, screening for APAD in hypertensive patients, and subsequent CV protective management, might be more beneficial. This assumption regarding screening for APAD is also supported in the literature [[41], [42], [43]].

In diabetes, treatment recommendations are essentially the same as for any vascular disease manifestation, with the exception for antiplatelet therapy [[44], [45], [46]], and almost all patients with diabetes should be recommended statin treatment to reduce their risk of CV-events [47,48]. Our finding that a pathological ABI worsens CV outcome for diabetic subjects supports other studies and enforces the notion that subjects with APAD and diabetes needs optimized treatment [49,50].

There is still a need for evaluation of screening benefits for APAD in terms of reduction of all‐cause mortality and CV events [13,15].

## 5 Limitations

One limitation in this study is that the registers used for follow-up only covers hospital-based activities, and not primary care. This is, however, likely to be a minor problem because few events occur outsides hospitals. Non-participation is another possible limitation and may have reduced the validity of the findings in the oldest age-group. This because elderly and severely ill subjects constituted the majority of non-participants. Effects of potential drug usage during the study period was neither observed nor analyzed. In this study, we chose to follow guideline recommendations for detection of PAD with ABI measurements and the threshold value was set to <0.9 for ABI, which catches about 90% of all PAD subjects. A limitation of this choice was the risk of misclassification of subjects with PAD and calcified vessels giving high ABI-results. In this cohort, the number of subjects with an ABI>1.4 was small and the influence of this group was not likely to alter our main results in an opposite direction.

## 6 Conclusion

APAD is well known to increase CV risk but its magnitude varies depending on this group's heterogeneity. For subjects with hypertension, diabetes mellitus and a smoking history, the addition of APAD contributes significantly to the age-adjusted CV risk, especially in women. The authors suggest further studies of screening activities for APAD with ABI-measurements in subjects with hypertension, diabetes or smoking habits for randomized controlled trials with treatment programs including statins.

## Conflict of interest

None.

## Financial support

The original study was supported by unrestricted grants from The 10.13039/501100003793Swedish Heart-Lung Foundation, Värmland's County Research Council, and Sanofi-Aventis® and Astra Zeneca®.

## Declaration of competing interest

The authors declare that they have no known competing financial interests or personal relationships that could have appeared to influence the work reported in this paper.
